# The accuracy of presepsin for the diagnosis of sepsis from SIRS: a systematic review and meta-analysis

**DOI:** 10.1186/s13613-015-0089-1

**Published:** 2015-12-08

**Authors:** Zhongjun Zheng, Libing Jiang, Ligang Ye, Yuzhi Gao, Luping Tang, Mao Zhang

**Affiliations:** Department of Emergency Medicine, Second Affiliated Hospital, School of Medicine and Institute of Emergency Medicine, Zhejiang University, No 88, Jiefang Rd, Hangzhou, 310009 China

**Keywords:** Sepsis, SIRS, Presepsin, sCD14-ST

## Abstract

**Background:**

Sepsis is a common condition that has a high mortality and morbidity that need prompt diagnosis and treatment. Biomarkers like Soluble CD14 subtype (sCD14-ST, presepsin) may be useful in identifying patients with sepsis and its diagnostic superiority has been confirmed by several preliminary studies. The aim of this study was systematically and quantitatively to evaluate the value of presepsin for the diagnosis of sepsis through the method of meta-analysis.

**Methods:**

Four major databases, including MEDLINE, EMBASE, ISI Web of Knowledge, and the Cochrane Library were systematically searched from inception to March 2015. Two investigators conducted the processes of literature search, study selection, data extraction, and quality evaluation independently. And the original data were extracted from all eligible individual studies to construct two-by-two tables.

**Results:**

A total of eight studies comprising 1757 patients were included in this meta-analysis. The pooled sensitivity, specificity, and diagnostic odds ratio were 0.77 (95 % confidence interval [CI]: 0.75–0.80), 0.73 (95 % CI 0.69–0.77), and 14.25 (95 % CI 8.66–23.42), respectively. The summary receiver operating characteristic curve (SROC) area under the curve (AUC) was 0.8598. The subgroup analysis based on excluding the outliers showed that the pooled sensitivity and specificity were 0.85 (95 % CI 0.81–0.89) and 0.65 (95 % CI 0.59–0.70), respectively. The AUC was 0.8213 with no significant heterogeneity.

**Conclusions:**

Presepsin has moderate diagnostic capacity for the detection of sepsis. Further research of presepsin is needed before widespread use in emergency department. And presepsin in combination with other laboratory biomarkers in diagnosing sepsis may be the focus of future studies.

## Background

Sepsis is a condition in which the immune system overreacts to infection, releasing inflammatory mediators into the peripheral blood and triggering widespread inflammation. Early detection and timely treatment of the above pathologic processes may be able to prevent the occurrence of multiple organ dysfunction (MODS) induced by severe sepsis and septic shock. Sepsis occurs in 1–2 % of all hospitalizations, and it is the leading cause of mortality in critically ill patients [1, 2]. Despite modern antibiotic therapy in combined with cardiovascular and respiratory support, mortality rates still remain between 30 and 60 % [3–5]. Therefore, early diagnosis and treatment are hot topic among intensivists [5].

However, rapid and accurate diagnosis of sepsis is often difficult in routine clinical practice because the clinical manifestations of this condition can overlap with many non-infectious causes of systemic inflammation, such as pancreatitis, ischemia, multiple trauma, and hemorrhagic shock, which are collectively termed a systemic inflammatory response syndrome (SIRS) [6]. Microbiological culture is considered as the reference standard in diagnosing infectious condition, whereas it usually needs several days to obtain the results [7].

Presepsin, also named soluble cluster-of-differentiation 14 subtype (sCD14-ST), is a 13 kDa protein that is a truncated N-terminal fragment of CD14 [8]. CD14 is a high-affinity receptor for complexes of lipopolysaccharide (LPS) and LPS-binding proteins (LPB), which is a multifunction cell surface glycoprotein expressed on the surface of various cells including monocytes, macrophages, neutrophils, and B cells [9, 10]. By shedding of CD14 from the cell membrane and releasing into circulation during infection status, the LPS–LPB–CD14 complex yields soluble CD14. Then, sCD14 is activated by plasma proteases during the circulating process, and thus generates a 13 kDa protein named sCD14-ST or presepsin. Presepsin increases significantly in the blood of sepsis patients, and it has been studied as a marker to differentiate sepsis from other non-infectious causes of SIRS [8]. The results of several early studies were encouraging, and it has been reported that the diagnostic value of presepsin was superior to procalcitonin (PCT) and C-reactive protein (CRP) [11, 12]. However, few studies produced disappointing results [13, 14]. Meanwhile, many studies included patients who did not have SIRS. This may add further uncertainty in assessing the diagnostic accuracy of presepsin. Thus, the aim of our study was to systematically explore the diagnostic value of presepsin in patients with sepsis.

## Methods

The present meta-analysis was conducted and reported according to the Preferred Reporting Items for Systematic Reviews and Meta-analyses Statement (PRISMA).

### Data sources

MEDLINE, Excerpta Medica database (EMBASE), ISI Web of Knowledge, and Cochrane Library databases were searched from the inception to March 2015. The following keywords or medical subject headings(MeSH) were used: “presepsin” or “sCD14-ST” or “soluble CD-14 subtype” or “soluble cluster of differentiation 14 subtype” and “sepsis” or “SIRS” or “systemic inflammatory response syndrome” or “infection.” We also searched the abstracts that were presented at the annual meetings of the American College of Emergency Physicians, the Society of Critical Care Medicine, and the Society for Academic Emergency Medicine. The reference lists of eligible articles and related reviews were also screened to identify further studies.

### Eligibility criteria

Two reviewers (ZJZ) and (LBJ) independently evaluated the studies for their eligibility to be included. In cases of disagreement, a consensus was reached by discussion or by consultation to a third reviewer (LGY). Studies were considered eligible if the following criteria were met: providing the presepsin concentrations of sepsis patients and non-sepsis patients; having sufficient data to construct the 2 × 2 contingency table; having a well-defined reference standard about diagnosing sepsis(defined by the American College of Chest Physicians/Society of Critical Care Medicine Consensus Conference, ACCP/SCCM) [15].

Reviews, correspondence, editorials, and conference abstracts were excluded. Studies with the same authors were cautiously investigated. Studies were also excluded if they were limited to restrictive subgroups, such as some special types of sepsis like burn sepsis. However, their reference lists were screened to identify further studies for inclusion.

### Data extraction

The following items were extracted using a specific sheet which was constructed in advance: authors and year of study, the country, study design, patient setting, number and characteristics of patients, presepsin measuring instrument, cut-off point, prevalence of sepsis, diagnostic sensitivity and specificity, inclusion criteria, and reference standard. Two reviewers independently extracted the data from each study. Disagreements were resolved by consensus.

### Quality assessment

The methodological quality of all individual study was assessed using the QUADAS-2 tool [16], which was recommended by the Cochrane handbook for diagnostic test accuracy reviews. The QUADAS-2 tool comprises four domains: patient selection, index test, reference standard, and flow and timing. Each domain is assessed in terms of risk of bias, and the first three domains are also assessed in terms of concerns regarding applicability. Signaling questions are included to help judge the risk of bias of each study. The assessment was performed independently by two reviewers (ZJZ, LBJ). Disagreements were resolved by consensus.

### Statistical analysis

True positive (TP), false positive (FP), false negative (FN), and true negative (TN) were obtained from each study. Sensitivity and specificity with its 95 % confidence were calculated from the 2 × 2 contingency table of each study. We added 1/2 to all cells of the studies with zero. Meanwhile, the meta-analysis was performed by calculating the pooled sensitivity, specificity, positive likelihood ratio (LR), negative LR, and diagnostic odds ratios (OR). Pooled results were constructed by using either the fixed-effect model when significant heterogeneity was absent or the random-effect model when significant heterogeneity was present [17].

Heterogeneity of the included studies was explored using the Cochrane Q test. Inconsistency (*I*^2^) expresses the variability attributable to heterogeneity across the studies in the form of a percentage. The spearman coefficient between the logit of sensitivity and logit of 1-specificity was performed to test threshold effect and a strong positive correlation indicates threshold effect [18]. There were several other factors that might contribute to the heterogeneity, including patient setting, characteristics of patients, presepsin measuring instrument, inclusion criteria, and reference standard [19]. *I*^2^ > 50 % or *P* < 0.05 suggested the presence of significant heterogeneity among included studies [20]. If substantial heterogeneity was found, the meta-regression techniques would be used to explore the reasons for the heterogeneity. Meta-regression was made using a generalization of Littenberg and Moses Linear model. The model was weighted by inverse of the variance or study size [18, 19]. In addition, subgroup analyses were performed according to the result of meta-regression.

Finally, if the studies were reasonably homogeneous, an AUC was calculated. The closer that the value of the area under the curve is to 1, the better validated the diagnostic test is. What’s more, we used a Q* point from the SROC curve to obtain the maximum joint sensitivity and specificity. The Q* point is the intersection between a symmetrical SROC curve and the antidiagonal line, at which sensitivity equals specificity. Comparing to other parameters, Q* point is a single-number summarizing of the test performance and has the advantage of being less affected by heterogeneity [18, 21].

Publication bias was tested by Deeks’ asymmetry test and a funnel plot. The slope coefficient with *P* < 0.05 indicated the presence of publication bias.

All analyses including the pooling of sensitivity, specificity, positive LR, negative LR, diagnostic OR, SROC curve, and meta-regression were conducted using freeware Meta-Disc, version 1.4 (Ramon Y Cajal Hospital, Madrid, Spain) [22]. Study quality was performed using Review Manager 5.3 (Oxford, UK: The Cochrane Collaboration). Publication bias was performed using Stata version 12.0 (Stata Corporation, College Station, Tex). By convention, *P* < 0.05 was considered statistically significant.

## Results

### Identification of studies

**Fig. 1:**
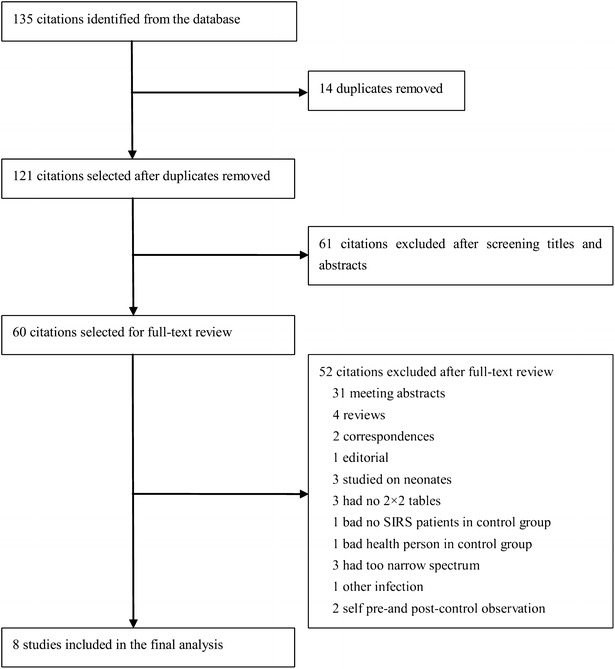
Identification, inclusion, and exclusion of the studies

The flow diagram of study selection is shown in Fig. 1. A total of 135 records were retrieved by searching the databases of MEDLINE, EMBASE, ISI Web of Knowledge, and Cochrane Library databases. After removing the 14 duplicates, the titles and abstracts of the remaining 121 records were screened. Then, 61 apparently irrelevant studies were excluded, and 60 potentially relevant articles were identified for further review.

After full-text review, 52 articles were excluded: 31 were Conference abstracts, four were review articles [23–26], two were correspondence letters [8, 27], one was editorial [28], three studied on neonates and had different reference standards [29–31], three did not have sufficient data to reconstruct the 2 × 2 contingency table [9, 32, 33], one had no SIRS patients in control group [34], one had health people in control group and it had different inclusion and exclusion criteria compared to our criteria [35], three had too narrow a spectrum of patients [36–38], one was about other infectious disease [39], and two studies were designed of self pre- and post-control observation [40, 41].

Finally, eight studies fulfilled the inclusion criteria and were eligible for our meta-analysis [7, 12, 13, 42–46]. The characteristics of all eight studies included in our analysis are shown in Table 1. A total of 1757 patients were included in our meta-analysis, of which, 1240 were emergency department visits. The prevalence of sepsis ranged from 16.37 to 85.11 %. All the studies used a compact automated immune analyzer PATHFAST (Japan or Germany), which is based on a chemiluminescent enzyme immunoassay, to determine the plasma presepsin concentrations. The test threshold of presepsin of diagnosing sepsis ranged from 317 to 729 pg/ml.

**Table 1:** Characteristics of the studies included (1757 patients)

Study	Year	Country	Patient setting	Mean age (years)	Patients (*n*)	Patients (type)	Measuring instrument	Cut-off (pg/ml)	Study design	Prevalence (%)	Sensitivity (%)	Specificity (%)	Reference standard
Endo et al.	2012	Japan	ED&ICU	NA	185	All type	PATHFAST,(Mitsubishi Chemical Medience Corporation, Japan)	600	PR&CR	62.16	87.80	81.40	ACCP/SCCM
Liu et al.	2013	China	ED	71.5	859	All type	PATHFAST,(Mitsubishi Chemical Medience Corporation, Japan)	317	PR&CR	79.16	70.80	85.80	ACCP/SCCM
Ulla et al.	2013	Italy	ED	64.4	189	All type	PATHFAST,(Mitsubishi Chemical Europe GmbH, Dusseldorf, Germany)	600	PR&CR	56.08	78.95	61.90	ACCP/SCCM
Behnes et al.	2014	Germany	ICU	67.9	96	All type	PATHFAST,(PROGEN Biotechnik GmbH, Germany; Mitsubishi Chemical Medience Corporation, Japan)	530	PR	84.38	90.00	60.00	ACCP/SCCM
Ishikura et al.	2014	Japan	ED	67.2	62	All type	PATHFAST,(Mitsubishi Chemical Medience Corporation, Japan)	647	PR&CR	69.35	93.00	76.30	ACCP/SCCM
Kweon et al.	2014	Korea	ED	64.01	93	All type(without burn patients)	PATHFAST,(Mitsubishi Chemical Medience Corporation, Japan)	430	PR&CR	78.49	87.70	82.20	ACCP/SCCM
Romualdo et al.	2014	Spain	ED	67.7	226	All type	PATHFAST,(Mitsubishi Chemical Europe GmbH, Dusseldorf, Germany)	729	PR&CR	16.37	81.10	63.00	ACCP/SCCM
Godnic et al.	2015	Slovenia	ICU	NA	47	All type	PATHFAST,(Mitsubishi Chemical Europe GmbH, Dusseldorf, Germany)	500	PR	85.11	84.60	62.50	ACCP/SCCM

*ICU* intensive care unit, *ED* emergency department, *PR* prospective recruitment, *CR* consecutive recruitment, *NA* not available, *ACCP/SCCM* American College of Chest Physicians/Society of Critical Care Medicine Consensus Conference

### Quality of studies

**Fig. 2:**
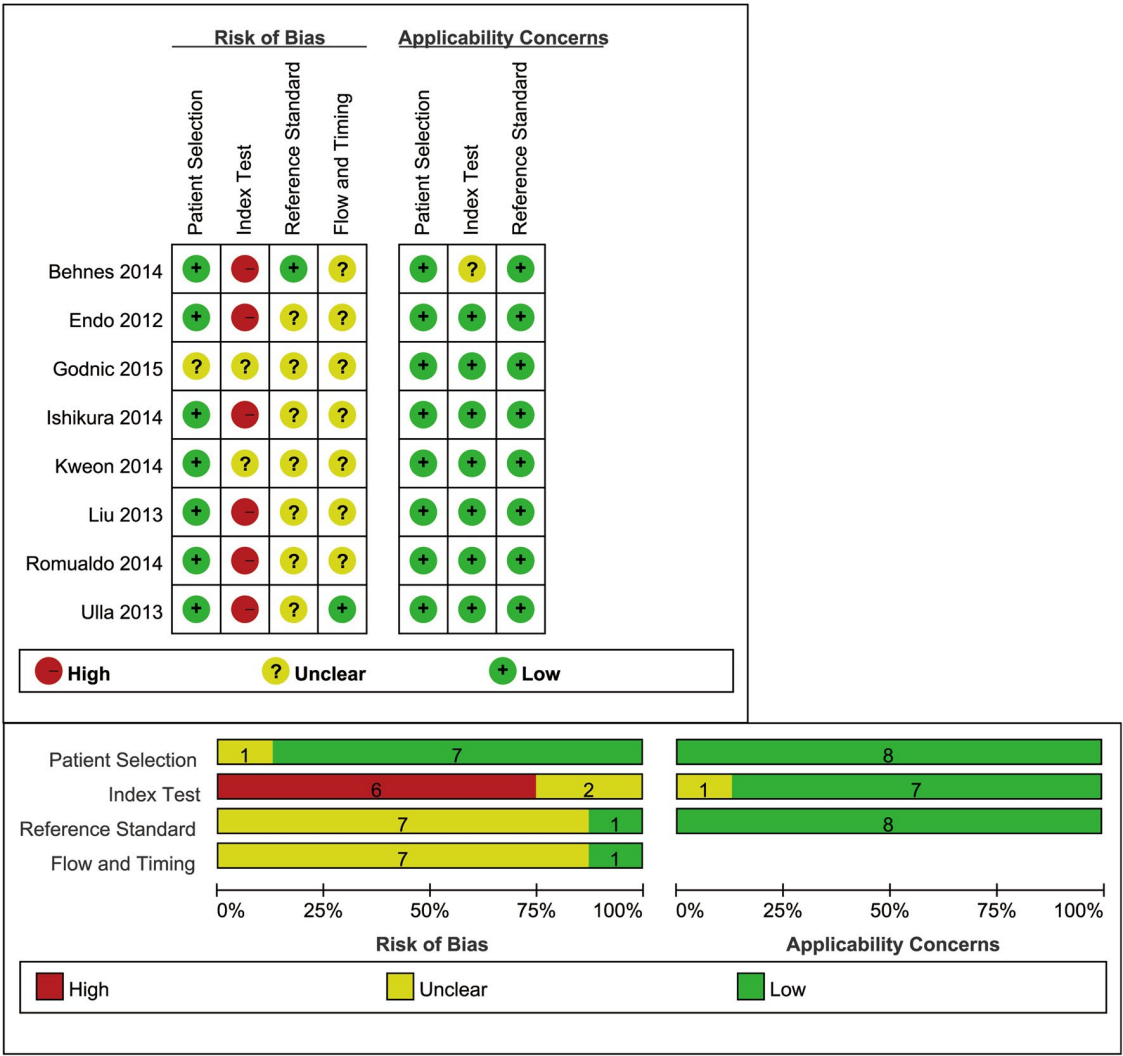
Risk of bias and applicability concerns

Details of the methodological assessment are shown in Fig. 2. We evaluated the quality of the eight included studies using the QUADAS-2 tool. According to the results of methodological assessment, all included studies possessed acceptable quality. The ACCP/SCCM guideline was used to diagnose sepsis in all studies, and the same index test criterion was applied to each patient. Meanwhile, all of them used a prospective study design. However, all the studies did not pre-specify a threshold except one [46]. There is no evidence that the reference standard results of the studies interpreted without knowledge of the results of the index test except one [44].

### Heterogeneity exploration and data synthesis

In the exploration of heterogeneity, the Spearman correlation coefficient between the logit of sensitivity and logit of 1-specificity was 0.190 (*P* = 0.651) with the slope of the regression line near zero, suggesting that there was no significant threshold effect present among the eight studies.

**Fig. 3:**
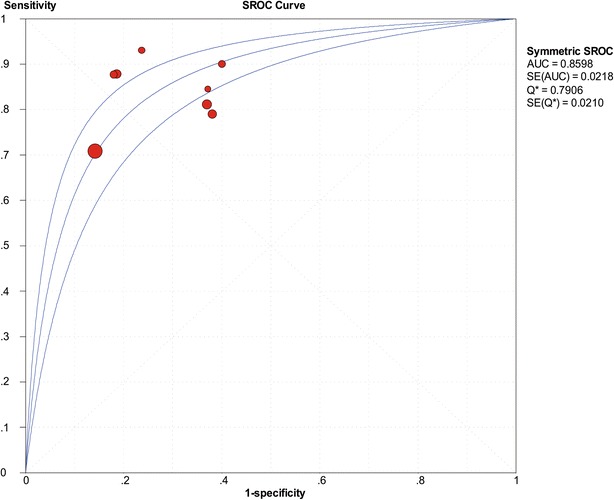
The SROC of presepsin for diagnosis sepsis

**Fig. 4:**
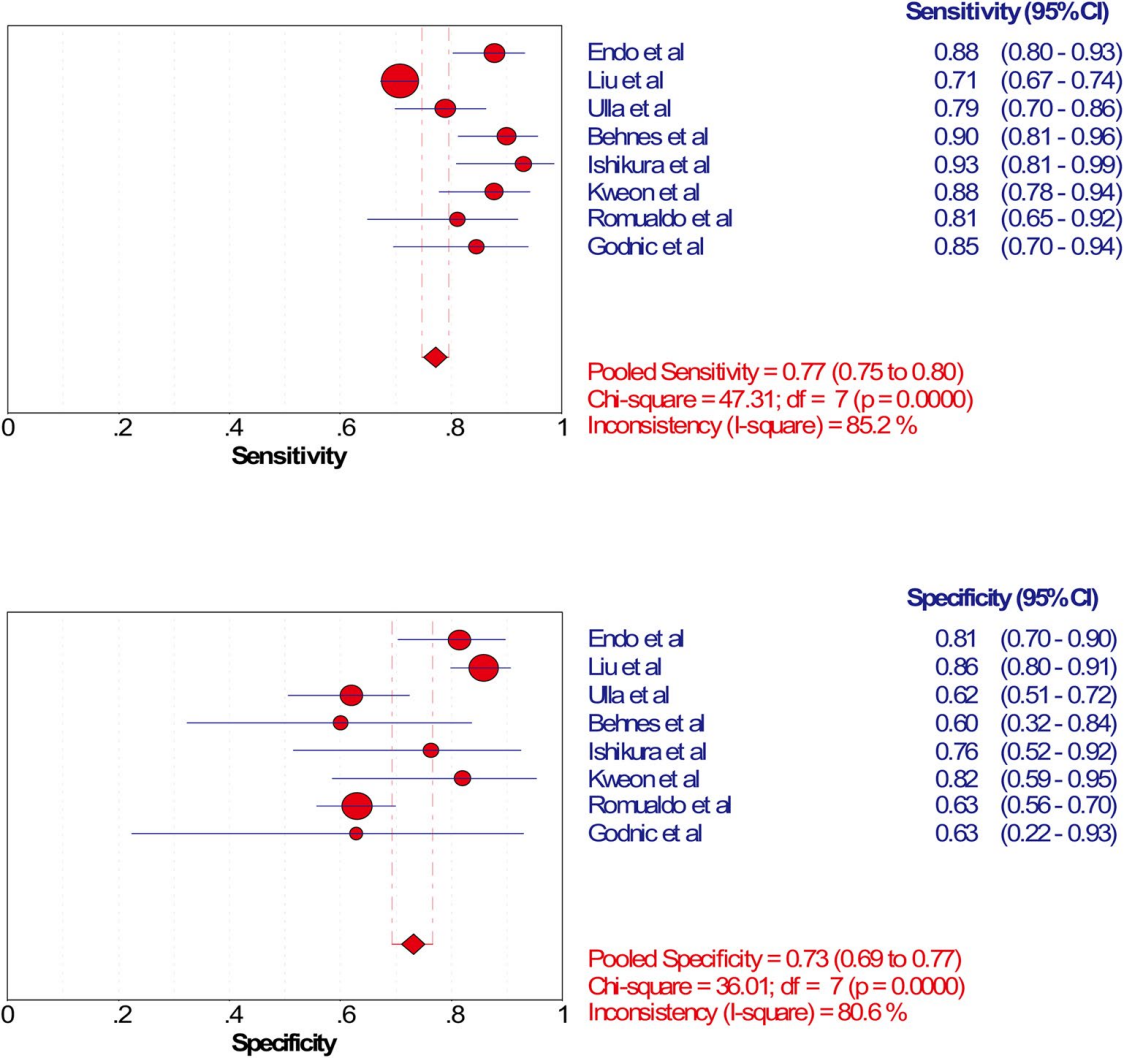
Forest plot of the pooled sensitivity and specificity

**Fig. 5:**
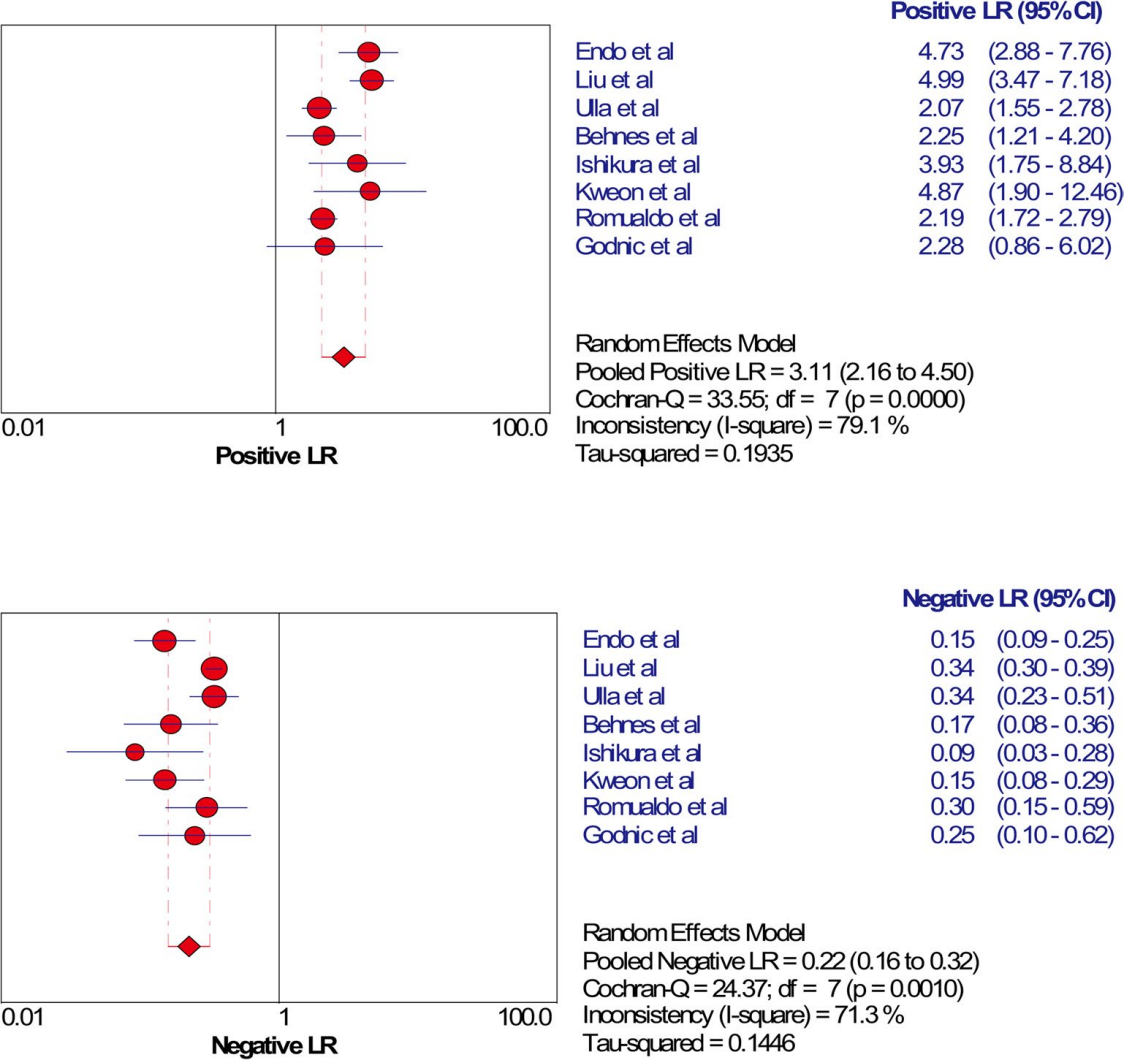
Forest plot of the pooled positive LR and negative LR

The SROC of presepsin is shown in Fig. 3, generating an AUC of 0.8598 (Q* = 0.7906). A random effects model was used to calculate the pooled mean difference and the 95 % confidence interval. The pooled diagnostic odds ratio of the eight studies was 14.25 (95 % CI 8.66–23.42). The pooled sensitivity and pooled specificity were 0.77 (95 % CI 0.75–0.80) and 0.73 (95 % CI 0.69–0.77), respectively (Fig. 4). The pooled positive LR and pooled negative LR were 3.11 (95 % CI 2.16–4.50) and 0.22 (95 % CI 0.16–0.32), respectively (Fig. 5). However, substantial degree of heterogeneity was observed in the summary estimates.

### Meta-regression

Since obvious heterogeneity was observed, the meta-regression technique was used to explore the heterogeneity other than threshold effect. The result is shown in Table 2. A substantial heterogeneity caused by the measuring instrument was found, and the subgroup analysis was performed by restricting studies to a similar measuring instrument (PATHFAST, Mitsubishi Chemical Medience Corporation, Japan).

**Table 2:** Meta-regression analysis for the possible sources of heterogeneity

Variances	Coefficient standard	Standard error	*P* value	RDOR	95 % CI
Inverse variance weights 1					
Cte	3.344	0.9147	0.0353	–	–
S	0.228	0.083	0.5130	–	–
Setting	0.341	0.2697	0.2952	1.41	0.60–3.32
Instrument	−0.945	0.4349	0.1180	0.39	0.10–1.55
Cut-off	0.046	0.0938	0.6558	1.05	0.78–1.41
Inverse variance weights 2					
Cte	3.560	0.8027	0.0114	–	–
S	0.282	0.2884	0.3838	–	–
Setting	0.295	0.2531	0.3082	1.34	0.67–2.71
Instrument	−0.978	0.4297	0.0851	0.38	0.11–1.24
Inverse variance weights 3					
Cte	4.244	0.5481	0.0006	–	–
S	0.458	0.2458	0.1215	–	–
Instrument	−1.200	0.3856	0.0265	0.30	0.11–0.81

The RDOR means the DOR for studies that lacked a particular methodologic feature divided by the DOR for studies without the flaw

—, not available, *Cte* constant term in the equation, *RDOR* relative diagnostic odds ratio, *S* indicator of threshold

*P* < 0.05 indicated the significant relationship between the characteristics of studies and the diagnostic odds ratio

**Fig. 6:**
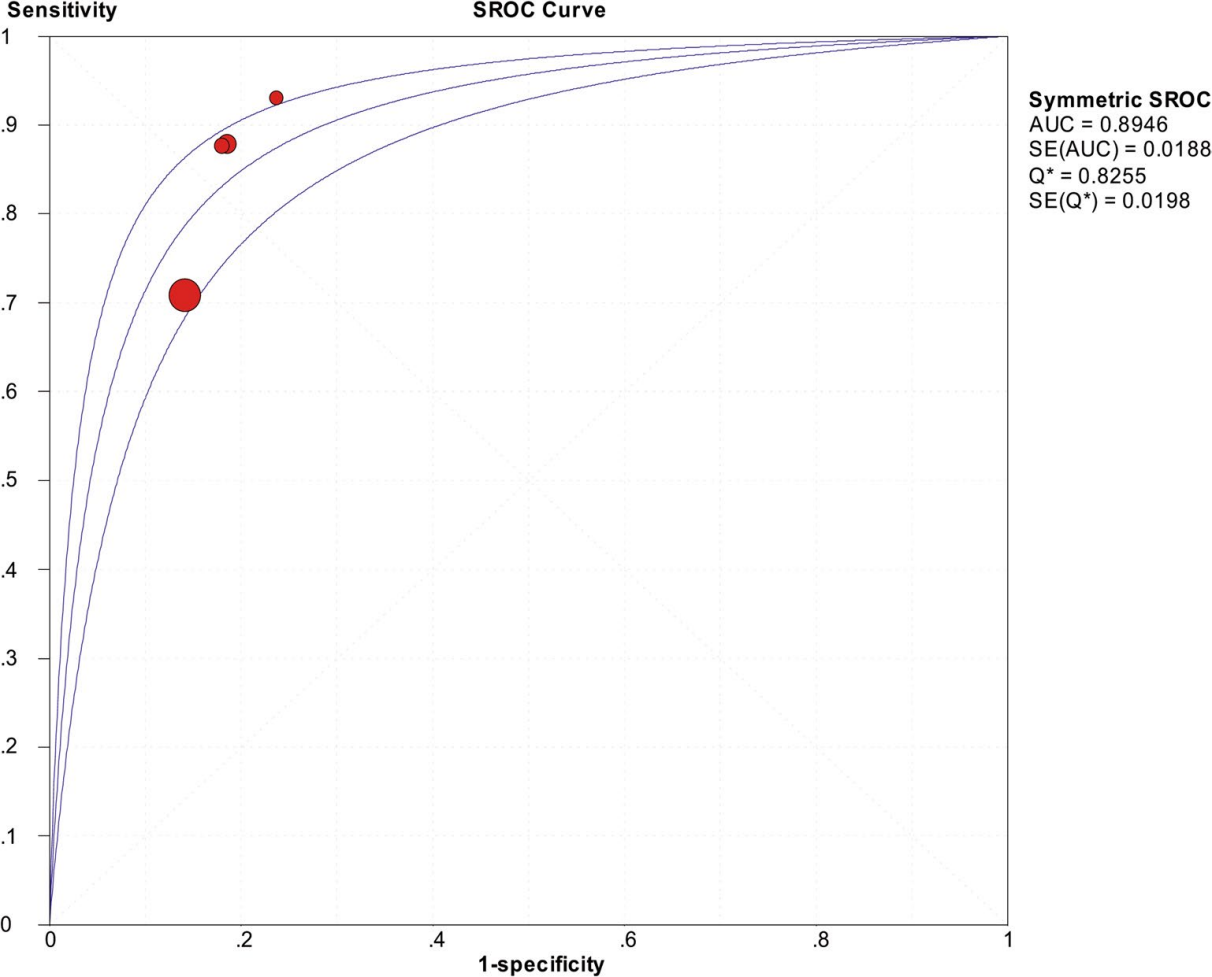
The SROC of presepsin for diagnosis sepsis based on meta-regression

The analysis included four studies [7, 12, 42, 43],the SROC is shown in Fig. 6, and the AUC was 0.8946 (Q* = 0.8255). The pooled sensitivity (random-effect model) and pooled specificity (fixed-effect model) were 0.75 (95 % CI 0.72–0.78) and 0.84 (95 % CI 0.79–0.88), respectively. The pooled positive LR (fixed-effect model) and pooled negative LR (random-effect model) were 4.82 (95 % CI 3.69–6.30) and 0.18 (95 % CI 0.09–0.35), respectively. The pooled diagnostic odds ratio was 18.57 (95 % CI 12.98–26.56). The result is shown in Table 3. However, there was still heterogeneity in pooled sensitivity and pooled negative LR (*I*^2^ = 91.4 % & *I*^2^ = 86.8 %).

**Table 3:** Meta-regression analysis based on restricting studies to a similar measuring instrument

Pooled results	Value	95 % CI	*P* value	*I* ^2^ (%)
Sensitivity	0.75	0.72–0.78	0.0000	91.4
Specificity	0.84	0.79–0.88	0.6635	0.0
Positive LR	4.82	3.69–6.30	0.9627	0.0
Negative LR	0.18	0.09–0.35	0.0000	86.8
Diagnostic OR	18.57	12.98–26.56	0.2197	32.1

### Subgroup analysis

**Fig. 7:**
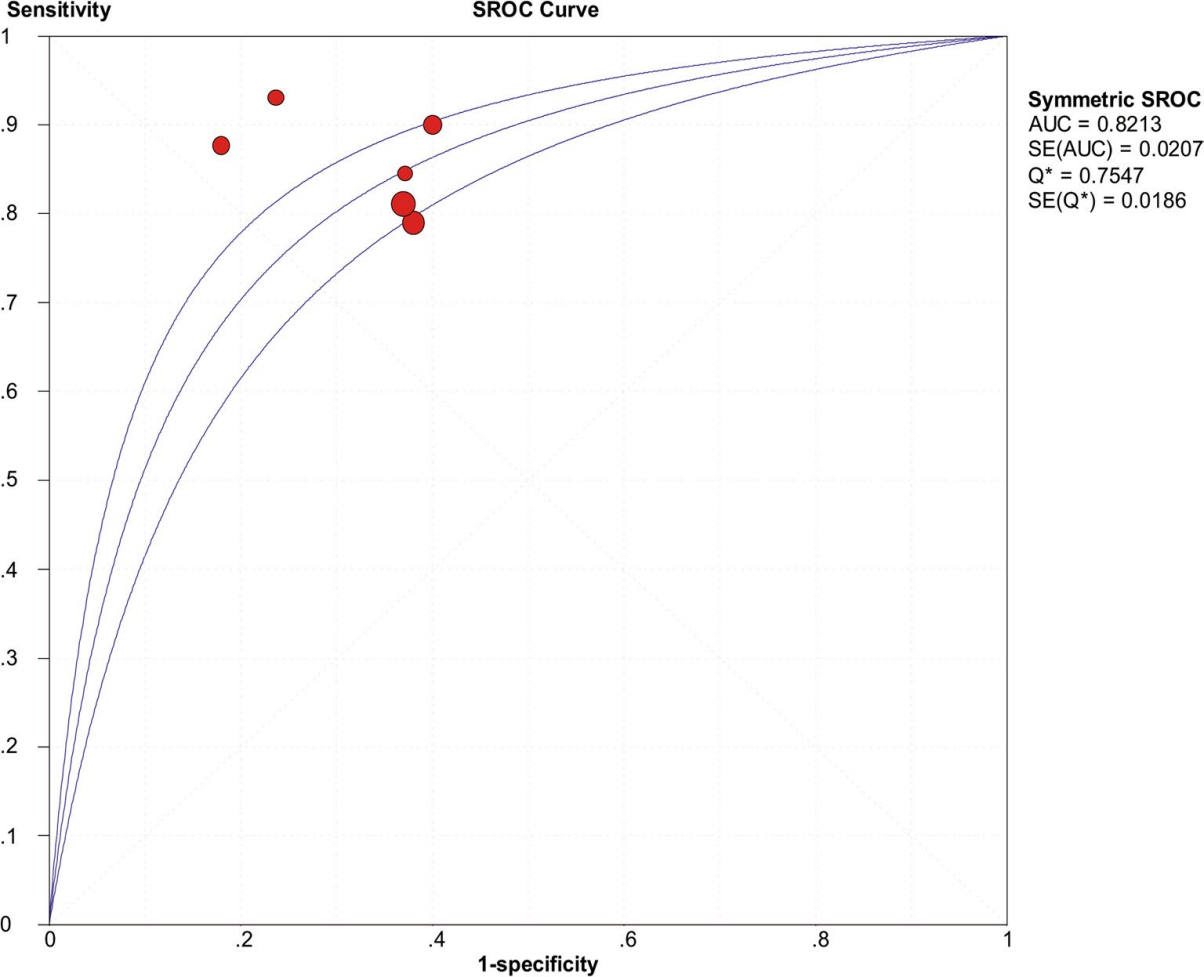
The SROC of presepsin for diagnosis sepsis based on excluding outliers

By observing the forest plots, the studies by Liu et al. and Endo et al. were found to be outliers and may account for most of the heterogeneity. After excluding these two studies, the heterogeneity diminished significantly. Subgroup analysis based on the rest of studies was performed with a fixed effects model, and the results are shown in Table 4. The pooled sensitivity, specificity, positive LR, negative LR, and diagnostic OR were 0.85 (95 % CI 0.81–0.89), 0.65 (95 % CI 0.59–0.70), 2.46 (95 % CI 2.03–2.98), 0.25 (95 % CI 0.19–0.33), and 9.47 (95 % CI 6.38–14.05), respectively. The SROC of these six studies are shown in Fig. 7 and the AUC was 0.8213 (Q* = 0.7547).

**Table 4:** Subgroup analysis based on excluding outliers

Pooled results	Value	95 % CI	*P* value	*I* ^2^ (%)
Sensitivity	0.85	0.81–0.89	0.1587	37.2
Specificity	0.65	0.59–0.70	0.4438	0.0
Positive LR	2.46	2.03–2.98	0.3472	10.7
Negative LR	0.25	0.19–0.33	0.1079	44.6
Diagnostic OR	9.47	6.38–14.05	0.1013	45.7

### Publication bias exploration

**Fig. 8:**
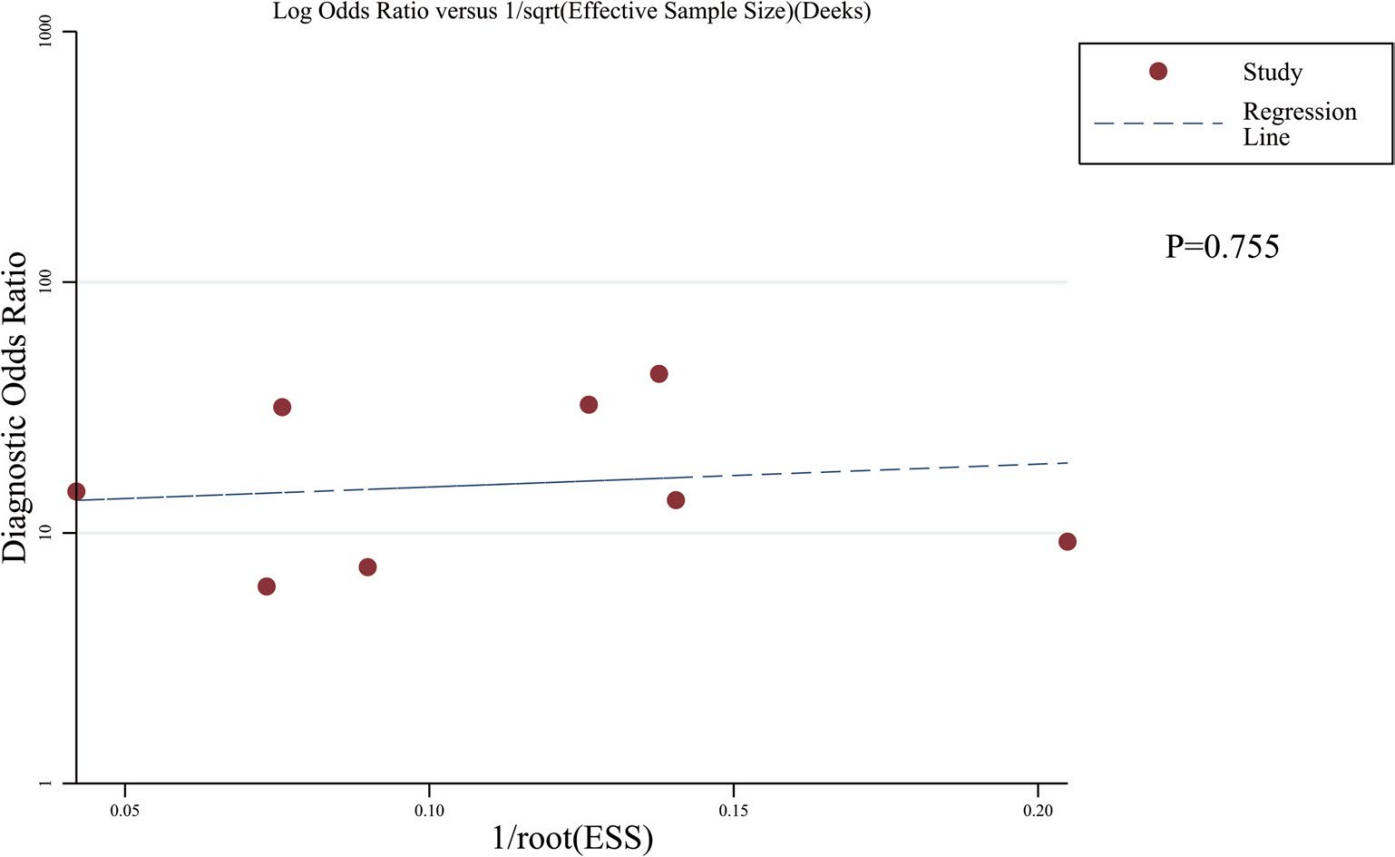
Funnel plot of publication bias (*P* = 0.755)

We used the Egger’s regression model to detect the publication bias, and the result is shown in Fig. 8. The Deeks’ funnel plot did not show significant asymmetry (*P* = 0.755), indicating that there was no significant publication bias in this meta-analysis.

## Discussion

The present systematic review and meta-analysis explored the accuracy of presepsin for the diagnosis of sepsis from SIRS patients. And the results showed that the biomarker presepsin had the acceptable pooled sensitivity (0.77) and pooled specificity (0.73). Accordingly, the AUC was 0.8598, indicating that the presepsin had a moderate diagnostic efficiency.

Sepsis causes millions of deaths globally each year and occurs in 1–2 % of all hospitalizations in the United States, affecting at least 750,000 persons and costing $17 billion per year to treat [1, 2]. As the high morbidity and mortality of sepsis, early diagnosis and treatment are essential to improve the prognosis of these patients [5]. However, due to the existence of other non-infectious causes of systemic inflammation (SIRS), it is critical to find a reliable biomarker to differentiate sepsis from SIRS at the early stage.

Various biological markers such as PCT, CRP, interleukins, and myeloid cells expressing triggering receptor-1 (TREM-1) have been reported as biomarkers in diagnosis with sepsis [47–49]. However, their clinical values are still controversial and uncertain except PCT. Meanwhile, the gold standard microbiological culture, which is used to distinguish infectious diseases from non-infectious conditions, lacks sensitivity and specificity, and there is often a substantial time delay [7].

As a new and promising biomarker, presepsin was first found in 2004 and has been revealed to be with superior diagnostic capacity when compared with other conventional diagnostic markers [11, 12]. Presepsin is a 13-kDa protein that is a fragment of CD14 with truncated N-terminal, the receptor for LPS/LBP complexes. It was identified as a protein whose levels increase specifically in the blood of sepsis patients. Vodnik et al. found that presepsin values were significantly higher in patients with sepsis (1508.3 ± 866.6 pg/mL) than the SIRS (430.0 ± 141.33 pg/mL) group (*P* < 0.0001) [38]. However, the results of few studies showed considerable differences, such as in the sensitivity range (0.71–0.93) and in the specificity range (0.60–0.86). Therefore, it is important for a meta-analysis to evaluate the value of a single laboratory test to identify patients at increased risk of sepsis from independent studies.

The meta-analysis presented above showed no threshold effect and significant publication bias. In addition, meta-regression was performed and a substantial heterogeneity caused by the measuring instrument was found. Therefore, subgroup analysis was performed by restricting studies to a similar measuring instrument (PATHFAST, Mitsubishi Chemical Medience Corporation, Japan); however, there were still heterogeneity in pooled sensitivity and pooled negative LR (*I*^2^ = 91.4 % & *I*^2^ = 86.8 %). The forest plots implied that the study by Liu et al. has the lowest sensitivity and the highest specificity. After excluding this study, heterogeneity still existed in pooled specificity (*I*^2^ = 53.7 %) and pooled positive LR (*I*^2^ = 60.7 %). The results in Fig. 4 showed that the study by Endo et al. may account for most of the heterogeneity in pooled specificity (*I*^2^ = 53.7 %) and pooled positive LR. Statistical pooling of the summary estimates was made after outliers excluded and the heterogeneity was eliminated, generating the acceptable pooled sensitivity (0.85) and pooled specificity (0.65).

The results of our study indicate moderate diagnostic performance of presepsin as a single test for diagnosing sepsis, which precludes recommendation for the routine use of sepsis as a screening or confirmatory test for sepsis. We are encouraged about the potential use of presepsin in combination with other clinical or laboratory markers in identifying high-risk patients, although we cannot make conclusions about this scenario because this was not an objective of the present study. The results of the study by Sargentini et al. confirmed the importance of monitoring a combination of several biomarkers in order to obtain a reliable diagnosis [35].

Because of its retrospective approach, any meta-analysis is prone to bias; however, we took several steps to minimize its impact. First, the selection of studies, extraction of data, and assessment of study quality were performed by two reviewers independently. Second, our study was adhered to the PRISMA statement. Third, only the ACCP/SCCM criterion was considered as our reference standard and studies were excluded if limited to restrictive subgroups. What is more, meta-regression and subgroup analysis were performed in this systematic review and meta-analysis, which made the result of the study more stable and reliable.

In addition, several limitations of this study should be put forward. First, studies included in our meta-analysis varied substantially in presepsin diagnostic criteria. Second, a limited number of studies were included in our meta-analysis. Third, the quality of all included studies varies substantially and might also influence the results of our studies. Therefore, further studies about presepsin for diagnostic assessment are need to be investigated, both in larger, multi-center studies and in prospective clinical studies, with sepsis patients without limited to restrictive subgroups.

## Conclusion

Our meta-analysis found that the presepsin test has moderate diagnostic ability for the detection of sepsis. On the basis of diagnostic accuracy, our data suggest that, before widespread clinical use, further research is needed of the presepsin test as a diagnostic test used in isolation to include or exclude sepsis in emergency department. And presepsin in combination with other laboratory biomarkers in diagnosing sepsis may be the focus of future studies.

## Abbreviations

Presepsin, sCD14-ST: cluster-of-differentiation 14 subtypes
CI: confidence interval
SROC: the summary receiver operating characteristic curve
AUC: the area under the receiver operating characteristic curve
MODS: multiple organ dysfunction
SIRS: systemic inflammatory response syndrome
LPS: lipopolysaccharide
LPB: lipopolysaccharide-binding proteins
PCT: procalcitonin
CRP: C-reactive protein
PRISMA: Preferred Reporting Items for Systematic Reviews and Meta-analyses Statement
EMBASE: Excerpta Medica database
MeSH: medical subject headings
ACCP/SCCM: American College of Chest Physicians/Society of Critical Care Medicine Consensus Conference
TP: true positive
FP: false positive
FN: false negative
TN: true negative
LR: likelihood ratio
OR: odds ratios
I^2^: inconsistency
TREM-1: myeloid cells expressing triggering receptor-1
